# Dietary Patterns in Toddlers with Excess Weight. the 2016 Pitnuts Study

**DOI:** 10.34763/devperiodmed.20172103.272285

**Published:** 2017-10-28

**Authors:** Halina Weker, Marta Barańska, Agnieszka Riahi, Małgorzata Strucińska, Małgorzata Więch, Grażyna Rowicka, Hanna Dyląg, Witold Klemarczyk, Agnieszka Bzikowska, Piotr Socha

**Affiliations:** 1 Nutrition Department, Institute of Mother and Child, Warsaw, Poland; 2 Human Nutrition Department, Faculty of Health Sciences, Medical University of Warsaw, Warsaw Poland; 3 Early Psychological Intervention Department, Institute of Mother and Child, Warsaw, Poland; 4 Gastroenterology, Hepatology, Nutrition Disorders and Paediatric Department, Children’s Memorial Health Institute, Warsaw, Poland

**Keywords:** children aged 1-3 years, excess weight, dietary patterns, dzieci w wieku 1-3 lata, nadmiar masy ciała, wzory żywienia

## Abstract

**Introduction:**

Children’s appropriate dietary pattern determines their optimal development, reduces the risk of childhood diseases and the risk of diet-dependent diseases, including obesity in adulthood.

**Aim:**

To analyze the dietary patterns of children with excess weight aged 1-3 years in comparison with the main components of the safe nutrition model including: the organization of meals (frequency of meals), selection of products (food intake), energy and nutritional value of children’s diets.

**Material and methods:**

The study was carried out in 2016 on a representative nationwide sample of children aged 5-36 months (n=1059). The analysis of dietary patterns covered 173 with excess weight children aged 13-36 months (BMI-z-score >1 SD). Their nutritional status was evaluated based on BMI and its standardisation according to the WHO reference child growth standards for children aged 0-5 years (BMI z-score). The diets of children were assessed using 3-day dietary records. The dietary patterns of the children who were analysed were determined using the cluster analysis (k-means method), including 11 variables concerning average daily intake of main food group products (cow’s milk, junior formula, milk products, bread, groats and rice, cereals, cured meats, fats, sugar and sweets, fruits, nectars and juices).

**Results:**

Three clusters of overweight and obese children with different dietary patterns were identified. The diet of children from the first cluster (n=58) was based primarily on junior formula and foods for infants and toddlers. This dietary pattern was defined as the “baby food diet”. The second cluster comprised 33 children whose diets were characterised by high consumption of cow’s milk and dairy products, as well as cereal products, including bread, groats, rice and breakfast cereals. This dietary pattern was defined the “milk and cereals diet”. The third cluster consisted of 82 children whose dietary pattern was characterised by high consumption of bread, cold meats and fats, sweets, juices and fruits (the “sandwich and sugar diet”). In all the clusters the average intake of vegetables and fruit by children with excess weight was significantly lower than the recommended amounts. The study showed too high intake of energy, protein, sodium, B vitamins and saccharose and an insufficient supply of calcium, fibre, vitamin D, vitamin E, LCPUFA, iodine and potassium in the children’s diet in reference to nutritional recommendations. Younger children with the “baby food diet” pattern, due to the contribution of enriched food, had a more balanced diet in relation to the model of safe nutrition (nutritional norms). Older children’s diets – in the third year of life, were characterized by a diversified choice of products that are a source of protein and carbohydrates (milk, breakfast cereals, meat, bread, cold meats, sugar from beverages, dairy desserts and juices).

**Conclusion:**

The identified dietary patterns of toddlers with excess weight differ from the safe nutrition model in terms of product selection and nutrient profile.

## Streszczenie

**Wprowadzenie:**

Prawidłowy wzór żywienia dziecka warunkuje jego optymalny rozwój, obniża ryzyko rozwoju chorób wieku dziecięcego, a w dorosłości chorób dietozależnych, w tym otyłości.

**Cel:**

Analiza wzorów żywienia dzieci w wieku 1-3 lata z nadmiarem masy ciała w odniesieniu do głównych składowych modelu bezpiecznego żywienia obejmujących organizację posiłków (częstość spożycia), dobór produktów (spożycie żywności), wartość energetyczną i odżywczą diet dzieci.

**Materiał i metody:**

Badanie przeprowadzono w 2016 r. na reprezentatywnej ogólnopolskiej próbie dzieci w wieku 5-36 miesięcy (n=1059). Do analizy wzorów żywienia włączono 173 dzieci w wieku 13-36 miesięcy z nadmiarem masy ciała (BMI-z-score >1 SD). Ich stan odżywienia oceniono na podstawie wskaźnika BMI wystandaryzowanego do referencyjnych siatek rozwoju dzieci 0-5 lat WHO (BMI z-score). Do oceny sposobu żywienia wykorzystano 3-dniowe zapisy diet dzieci. Wzory żywienia dzieci z nadmiarem masy ciała określono za pomocą analizy skupień (metoda k-średnich), do której włączono zmienne (n=11) dotyczące średniego dziennego spożycia mleka i przetworów mlecznych (w tym mleka krowiego, mleka modyfikowanego, mlecznych produktów – jogurty, deserki), produktów zbożowych (pieczywa, kasz i ryżu, płatków śniadaniowych), a także wędlin, tłuszczów, cukru i słodyczy, owoców, nektarów oraz soków.

**Wyniki:**

Wyodrębniono trzy grupy dzieci z nadmiarem masy ciała różniących się wzorami żywienia. W grupie pierwszej (n=58) podstawą żywienia dzieci było mleko modyfikowane oraz gotowa żywność przeznaczona dla niemowląt i małych dzieci. Ten wzór żywienia dzieci określono jako „baby food diet”. W grupie drugiej znalazło się 33 dzieci, które spożywały głównie mleko krowie, przetwory mleczne, produkty zbożowe (pieczywo, kasze, ryż i płatki). Ten wzór żywienia określono jako „milk and cereals diet”. W grupie trzeciej znalazło się 82 dzieci, których wzór żywienia charakteryzował się wysokim spożyciem pieczywa, wędlin i tłuszczu, a także słodyczy, soków i owoców („sandwich and sugar diet”). Średnie spożycie warzyw i owoców przez dzieci z nadmiarem masy ciała, we wszystkich skupieniach, było zdecydowanie niższe od zalecanych ilości. W przeprowadzonym badaniu w dietach większości dzieci stwierdzono nadmiar energii, białka, sodu, a także witamin z grupy B i sacharozy oraz niedobór – wapnia, błonnika, wit. D, E, a także DWKT, jodu i potasu, w odniesieniu do zaleceń (normy żywienia). Dzieci młodsze o wzorze żywienia „baby food diet”, z uwagi na udział żywności wzbogacanej (np. mleko modyfikowane, kaszki) miały korzystniej zbilansowaną dietę w odniesieniu do modelu bezpiecznego żywienia (normy żywienia). Diety dzieci starszych – w trzecim roku życia, charakteryzowały się zróżnicowanym doborem produktów będących źródłem białka i węglowodanów (mleko, płatki, mięso, pieczywo, wędliny, cukier ze słodkich napojów, deserów mlecznych i soków).

**Wnioski:**

Wyodrębnione wzory żywienia małych dzieci z nadmiarem masy ciała odbiegają od składowych modelu bezpiecznego żywienia w zakresie doboru produktów, jak i profilu składników pokarmowych.

## Introduction

Appropriate diet determines a child’s its optimal development, reduces the risk of childhood and diet-dependent diseases, including obesity in adulthood [1, 2, 3, 4]. In recent years, research proved that childhood obesity reflects interactions of genetic and environmental factors, including dietary ones, and that excessive weight in toddlers is a predictor of obesity at preschool and school age [1, 5, 6, 7]. It also increases the risk of chronic non-infectious diseases, such as type 2 diabetes or cardiovascular diseases. In obesity prevention, the basis for an appropriate diet is provided by safe nutrition models for toddlers, including recommendations for nutritional practices, choice of food in the diet, as well as nutrient profiles compliant with the standards [8, 9]. Therefore, dietary patterns of children, defined by the consumption of foods from various product groups and nutrient profiles, should be similar to the recommendations of the safe nutrition model.

## Aim

To analyse the dietary patterns of with excess weight children aged 1-3 years in comparison with the main components of the safe nutrition model including organization of meals (frequency of meals), selection of products (food intake), energy and nutritional value of children’s diets.

## Material and methods

The PITNUTS study was carried out in 2016 on a representative nationwide sample of children aged 5-36 months (n=1059). The analysis of dietary patterns covered 173 overweight and obese children aged 13-36 months (BMI-z-score>1SD and more). Their nutritional status was evaluated based on measurements of body weight and height, calculation of their BMI and its standardisation according to the WHO reference child growth standards for children aged 0-5 years (BMI z-score) in line with the applicable methodology [10]. The diets were assessed using 3-day dietary records of children prepared by their parents. The records were used for the estimation of daily food rations (food consumption) and the nutritional value of the rations was calculated using the Dieta 5.0 nutritional programme [11, 12, 13]. The data from the questionnaire on environmental and family conditions of the children who were analysed were also taken into account.

The dietary patterns of the children with excess weight analysed were determined using the cluster analysis (k-means method), incorporating 11 variables on average daily consumption of milk and dairy products (including cow’s milk, follow-up formula and fermented milk beverages), cereal products (bread, groats and rice, breakfast cereals), as well as fruits, cold meats, fats, sugar and sweets, nectars and juices.

In the clusters of children with various dietary patterns that were obtained, differences were analysed in terms of environmental variables (parents’ education, place of residence, socio-economic status and parents’ BMI) and nutritional variables (meeting dietary standards for energy, macronutrients, calcium and vitamin D, nutritional practices and consumption of recommended food rations, i.e. appropriate consumption of products from various food groups).

The statistical analysis of the results obtained was performed using the Statistica 12 PL statistical package.

The analyses were performed using the chi-square test (variables on a nominal scale) and the Kruskal-Wallis Anova rank test (variables on an ordinal or higher scale). The statistical significance level of p<0.05 was adopted.

## Results

The cluster analysis using the k-means method made it possible to distinguish three clusters of children with different dietary patterns.

The first cluster comprised 58 children in the second year of life (age median 19.9 months). The other two clusters included children in the third year of life (age median: 26.0 and 26.6 months).

The diet of the children from the first cluster (n=58) was based primarily on junior formula and ready-to-serve foods for infants and toddlers. This dietary pattern was defined as the “baby food diet”.

The second cluster comprised 33 children whose diets were characterised by a substantial share of cow’s milk and dairy products, as well as cereal products, including bread, groats, rice and breakfast cereals. The children from that group also ate a lot of cold meats and products containig sugar, including sweets. This dietary pattern was defined as the “milk and cereals diet”.

The third cluster consisted of 82 children whose dietary pattern was characterised by high consumption of bread, cold meats and fats, as well as a significant amount of products being a source of simple sugars and disaccharides (sweets, juices, fruit). The consumption of cow’s milk was reduced and partly replaced by sweet dairy products, including fruit yoghurts and milk desserts. The dietary pattern of the children from that cluster was defined as the “sandwich and sugar diet”.

Table I presents the characteristics of children in individual clusters in terms of their family background. Statistically significant differences in the parents’ education of the children from the analysed clusters were found (mothers p=0.09, fathers p=0.02). The parents of the children from the second cluster were the least educated. The analysed groups of children did not vary in terms of place of residence or subjective assessment of their financial situation. The BMIs of the parents of children from various clusters did not show statistically significant differences, either.

**Table I:** Baseline characteristics. Tabela I. Charakterystyka badanych dzieci.

Variables *Zmienne*	All children *Dzieci ogółem* (N=173)		Cluster 1 *Grupa 1* BABY FOOD DIET (N=58)		Cluster 2 *Grupa 2* MILK AND CEREALS DIET (N=33)		Cluster 3 *Grupa 3* SANDWICH AND SUGAR DIET(N=82)		P value
Age [months] *Wiek [miesiɋce]* median (1Q-3Q) *mediana (1Q-3Q)*	23.5 (17.8-28.8)		19.9 (15.4-24.4)		26.6 (20.0-28.8)		26.0 (19.0-30.1)		<0.0001^*^
Children's BMI z-score *BMI z-score dzieci* median (1Q-3Q) *mediana (1Q-3Q)*	1.6 (1.3-2.3)		1.6 (1.2-2.1)		1.8 (1.3-2.7)		1.6 (1.3-2.3)		0.5
Mothers' BMI z-score *BMI z-score matek* median (1Q-3Q) *mediana (10-30)*	23.4 (21.3-26.5)		22.9 (21.0-26.0)		22.9 (21.8-29.9)		23.8 (21.3-26.0)		0.7
Fathers' BMI z-score *BMI z-score ojców* median (1Q-3Q) *mediana (1Q-3Q)*	26.6 (24.7-28.9)		26.8 (24.5-29.4)		26.9 (24.6-29.4)		26.4 (24.7-28.0)		0.8
Parents' education *Wykształcenie rodziców*	Mothers [%] *Matki*	Fathers [%] *Ojcowie*	Mothers [%] *Matki*	Fathers [%] *Ojcowie*	Mothers [%] *Matki*	Fathers [%] *Ojcowie*	Mothers [%] *Matki*	Fathers [%] *Ojcowie*	0.09 (Mothers) 0.02^*^(Fathers)
Primary *Podstawowe*	18.6	31.4	15.5	*22.7*	34.3	53.5	14.7	29.3	
Secondary *Średnie*	36.0	37.2	34.5	39.6	31.3	28.6	39.0	38.7	
University *Wyższe*	45.4	31.4	50.0	37.7	34.4	17.9	46.3	32.0	
Place of residence [%] *Miejsce zamieszkania*									
Urban agglomerations *Aglomeracje*	20.8		25.9		21.2		17.1		0.3
Mid-sized cities *Miasta*	30.6		20.7		42.4		32.9		
Countryside *Wieś*	48.6		53.4		36.4		50.0		
Economic status [%] *Sytuacja materialna*									
Poor *Zła lub* *bardzo zła*	0.6		1.7		0.0		0.0		0.6
Average *Przeciętna*	41.6		41.4		45.4		40.2		
Good *Dobra lub bardzo dobra*	57.8		59.6		54.6		59.8		

Median *(1Q.-3Q)/mediana (1Q-3Q) -* median and interquartile *range/mediana i rozstęp kwartylny*

* StatisticaIly significant differences between three clusters of children *(p<0.05)/statystycznie istotne różnice pomiędzy trzema grupami dzieci*.

Table II presents the comparison of average daily food rations of children with different dietary patterns with respect to the recommended food ration for children aged 13-36 months [9].

**Table II:** Comparison of the average daily food rations of children with excess weight aged 1-3 years with different dietary patterns with regard to model food rations. Tabela II. Porównanie przeciętnej całodziennej racji pokarmowej dzieci w wieku 1-3 lata z nadmiarem masy ciała różniɋcych się wzorami żywienia w odniesieniu do zalecanej racji pokarmowej.

	Groups of food Products [g] *Grupy produktów [g]*	Recommended food ration for children aged 13-36 months *Zalecana racja Pokarmowa dla dzieci 13-36 miesięcy*	Daily food ration *Dzienna racja pokarmowa*				Children [%] with consumption of food exceeding recommendations *Odsetek dzieci ze spożyciem żywności powyżej zaleceń*			
			All children *Dzieci ogółem* (N=173) Median (1Q-3Q) *mediana (1Q-3Q)*	Cluster 1 *Grupa 1* BABY FOOD DIET (N=58) Median (1Q-3Q) *mediana (1Q-3Q)*	Cluster 2 *Grupa 2* MILK AND CEREALS DIET (N=33) Median (1Q-3Q) *mediana (1Q-3Q)*	Cluster 3 *Grupa 3* SANDWICH AND SUGAR DIET (N=82) Median (1Q-3Q) *mediana (1Q-3Q)*	All children *Dzieci ogółem* (N=173)	Cluster 1 *Grupa 1* BABY FOOD DIET (N=58)	Cluster 2 *Grupa 2* MILK AND CEREALS DIET (N=33)	Cluster 3 *Grupa 3* SANDWICH AND SUGAR DIET (N=82)
1.	Cereal products and potatoes *Produkty zbożowe i ziemniaki*									
	Bread^a^, ^b^ *pieczywo mieszone*	20	46.7 (26.7-63.3)	29.6 (11.7-50.0)	53.3 (43.3-65.0)	50.3 (30.0-70.0)	83.2	65.5	97.0	90.2
	Flour and pasta *mɋka, makarony*	25	19.7 (12.2-29.5)	17.7 (10.3-28.2)	21.6 (15.3-28.4)	20.6 (12.5-34.6)	34.7	31.0	36.4	36.6
	Groats, rice, breakfast cereals^a^, ^b^ *kasze, ryż, płatki śniadaniowe*	30	16.5 (8.0-32.2)	11.2 (3.7-28.9)	33.5 (10.8-49.5)	16.6 (9.3-27.3)	27.7	20.7	54.6	22.0
1A.	Potatoes *Ziemniaki*	100	76.0 (42.1-123.7)	68.9 (27.0-109.2)	81.9 (54.2-149.5)	81.7 (46.3-127.3)	37.6	34.5	42.4	37.8
2.	Vegetables and fruits ^a^, ^b^ Warzywa i owoce	450	271.6 (198.4-374.0)	259.3 (168.6-381.7)	225.4 (149.1-325.6)	295.0 (234.6-443.6)	15.0	10.3	3.0	23.2
	vegetables *warzywo*	200	111.1 (69.6-159.2)	99.0 (71.7-139.9)	100.2 (60.2-139.0)	121.7 (76.2-173.6)	10.4	8.6	3.0	14.6
	fruits^a^ *owoce*	250	154.7 (93.3-226.7)	134.6 (90.4-225.7)	120.8 (68.8-197.9)	183.1 (119.0-259.3)	20.2	17.2	9.1	26.8
3.	Milk and dairy products *Mleko i produkty mleczne*									
	milk and fermented milk beverages^a^, ^b^ *mleko i mleczne napoje fermentowane*	550	467.0 (343.4-666.3)	555.2 (418.4-699.9)	719.0 (592.3-867.1)	348.0 (227.5-467.0)	38.2	51.7	75.8	13.4
	incl. liquid milk^a^, ^b^ *w tym mleko płynne*	450	297.9 (15.8-446.7)	429.8 (321.7-492.6)	469.7 (377.5-584.5)	142.6 (66.4-232.9)	24.9	39.7	60.6	0.0
	cow's milk^a^ *mleko krowie*	--	111.2 (42.3-257.0)	38.6 (12.6-84.9)	468.6 (377.5-528.2)	118.7 (58.8-208.8)	--	--	--	--
	baby formula^a^ *mleko modyfikowane*	--	0.0 (0.0-278.3)	360.0 (278.3-450.0)	0.0 (0.0-0.0)	0.0 (0.0-0.0)	--	--	--	--
	fermented milk beverages *mleczne napoje fermentowane*	100	25.0 (0.0-56.9)	18.3 (0.0-53.3)	20.0 (0.0-50.0)	33.3 (0.0-76.7)	12.1	8.6	9.1	15.9
	curd cheese *sery twarogowe*	10-15	13.1 (1.2-45.7)	9.6 (0.0-33.3)	22.0 (1.7-50.0)	12.4 (2.9-50.0)	46.2	39.7	57.6	46.3
	rennet cheese *sery podpuszczkowe*	2	0.0 (0.0-5.7)	0.0 (0.0-5.0)	0.3 (0.0-8.3)	0.0 (0.0-6.7)	40.5	31.0	48.5	43.9
4.	Meat, cold meats, fish and eggs *Mięso, wędliny, ryby oraz jaja*									
	meat, poultry, cold meats^a^ *mięso, drób, wędliny*	20	(54.480.4 -114.6)	(48.3 70.3 - 100.2)	(66.086.4 -121.8)	(58.083.8 - 127.3)	94.8	93.1	93.9	96.3
	fish *ryby*	10	0.0 (0.0-4.7)	0.0 (0.0-0.0)	0.0 (0.0-19.2)	0.0 (0.0-9.3)	21.4	13.8	27.3	24.4
4A.	Eggs^a^, ^b^ *Jaja*	25	26.4 (9.3-42.1)	19.7 (7.9-33.3)	24.7 (8.8-42.1)	35.9 (11.8-46.3)	51.4	36.2	48.5	63.4
5.	Fats^a^, ^b^ *Tłuszcze*	16	15.6 (9.1-23.1)	10.5 (6.1-16.7)	21.3 (13.9-28.8)	17.2 (12.4-24.6)	47.4	25.9	63.6	56.1
6.	Sugar and sweets^a^, ^b^ *Cukier i słodycze*	20	25.3 (13.8-36.9)	19.3 (10.6-26.5)	34.8 (24.5-49.5)	29.0 (14.8-39.0)	61.3	48.3	78.9	63.4

Median (1Q-3Q)/med/ono *(1Q-3Q) -* median and interquartile *range/mediana i rozstęp kwartylny*

a Statistically significant differences in consumption of food products between the three clusters of children (KruskaI-Wallis rank Anova; *p<0.05)/Statystycznie istotne różnice w spożyciu produktów spożywczych pomiędzy trzema skupieniami dzieci (Anova rang Kruskala-Wallisa; p<0,05)*.

b Statistically significant differences in the odds of children from different clusters with consumption of food exceeding recommendations (chi2 test; *p<0.05)/Statystycznie istotne różnice w odsetkach dzieci z różnych skupień ze spożyciem żywności powyżej zaleceń (test chi2; p<0,05)*

The identified dietary patterns of children with excess weight were not compliant with the recommended daily food rations. Almost every child from the second and third cluster ate over twice as much bread and several times more meat and meat products as compared to the recommended daily food ration. The consumption of pasta and potatoes exceeding recommendations was observed in the diets of more than 30% of the children. About 50% of the subjects ate too many dairy products (cheese), eggs and fats. The quantity of sugar and sweets in the diets of 48.3-78.9% of the children exceeded daily limits. The odds of the children not following the sugar and sweet consumption limitations is increasing with the children’s age. The average intake of vegetables and fruits in all the clusters of children with excess weight was significantly lower than the recommended amounts.

Table III presents the energy and nutritional value of the identified dietary patterns of the children analysed in comparison with dietary standards [11]. The majority of diets of the children (in all clusters) exceeded the EAR standard for protein (100% of children) and digestible carbohydrates (86.2% children in the first cluster, 97.0% children in the second cluster, 87.8% children in the third cluster). A higher than recommended share of energy from saccharose was identified in the diets of 63.8-79.3% of the subjects.

**Table III:** Comparison of an average energy and nutrient intake in diets of children with excess weight aged 1-3 years with different dietary patterns with regard to nutritional recommendations. Tabela III. Porównanie przeciętnej wartości energetycznej i odżywczej diet dzieci w wieku 1-3 lata z nadmiarem masy ciała różniɋcych się wzorami żywienia w odniesieniu do zaleceń żywienia.

Energy and nutrients *Energia i składniki pokarmowe*	Nutritional recommendations EAR / Al^+^ *Normy żywienia EAR/AI^+^*	Nutritional value of children's diets *Wartość odżywcza diet dzieci*				Children [%] with energy and nutrient intake below EAR/AI *Odsetek dzieci ze spożyciem energii i składników pokarmowych poniżej normy EAR/AI*			
		All children *Dzieci ogołem* (N=173) Median (1Q-3Q) *mediana (1Q-3Q)*	Cluster 1 *Grupa 1* BABY FOOD DIET (N=58) Median (1Q-3Q) *mediana (1Q-3Q)*	Cluster 2 *Grupa 2* MILK AND CEREALS DIET (N=33) Median (1Q-3Q) *mediana (1Q-3Q)*	Cluster 3 *Grupa 3* SANDWICH AND SUGAR DIET (N=82) Median (1Q-3Q) *mediana (1Q-3Q)*	All children *Dzieci ogółem* (N=173)	Cluster 1 *Grupa 1* BABY FOOD DIET (N=58)	Cluster 2 *Grupa 2* MILK AND CEREALS DIET (N=33)	Cluster 3 / *Grupa 3* SANDWICH AND SUGAR DIET (N=82)
Energy [kJ]^a^ *Energia*	***--***	4885.6 (3979.7-5928.5)	4449.9 (3625.7-5117.6)	5854.1 (5076.2-6683.6)	4725.8 (3826.4-5804.5)	--	--	--	--
Energy [kcal]^a^, ^b^ *Energia*	1000	1168.5 (951.2-1414.3)	1063.2 (866.4-1222.9)	1391.2 (1210.6-1596.0)	1128.2 (914.1-1385.1)	31.2	41.4	6.1	34.2
Protein [g]^a^ *Białko*	12	43.0 (34.8-52.3)	37.2 (28.8-46.6)	56.7 (47.7-61.1)	43.0 (35.1-52.0)	0.0	0.0	0.0	0.0
Fat [g]^a^, ^b^ *Tłuszcz*	39	39.3 (30.9-51.7)	33.5 (29.7-43.4)	52.2 (42.0-57.2)	39.9 (30.3-46.3)	49.1	63.8	24.2	48.8
LCPUFA [g]^a^ *DWKT*	0.25+	0.0 (0.0-0.1)	0.0 (0.0-0.1)	0.1 (0.0-0.1)	0.0 (0.0-0.1)	91.9	98.3	87.9	89.0
Carbohydrates [g]^a^ *Węglowodany*	--	170.9 (131.4-197.3)	158.8 (125.3-186.1)	182.8 (166.0-219.4)	167.7 (127.2-196.9)	--	--	--	--
Digestible carbohydrates [g]^a^ *Węglowodany przyswajalne*	100	162.6 (123.6-187.4)	151.3 (117.7-178.2)	174.6 (154.6-209.2)	158.8 (119.4-183.4)	11.0	13.8	3.0	12.2
Saccharose [g]^a^ *Sacharoza*	--	38.8 (25.8-53.3)	30.9 (21.8-40.0)	48.3 (36.3-66.1)	41.3 (28.5-55.5)	--	--	--	--
Lactose [g]^a^ *Laktoza*	--	17.7 (9.7-27.6)	28.1 (21.2-35.5)	25.6 (22.1-32.4)	9.8 (7.0-14.0)	--	--	--	--
Starch [g]^a^ *Skrobia*	--	56.0 (44.7-79.2)	47.2 (33.8-62.7)	67.6 (49.9-84.6)	63.2 (48.4-83.1)	--	--	--	--
Fiber [g] *Błonnik*	10+	9.4 (7.5-12.1)	9.4 (7.5-11.7)	8.7 (7.6-10.2)	9.9 (7.5-12.7)	56.1	55.2	72.7	50.0
Energy from protein [%]^a^ *Energia z białka*	--	14.9 (13.3-16.7)	13.9 (12.8-15.2)	16.0 (14.8-17.2)	15.3 (13.3-17.3)	--	--	--	--
Energy from fat [%] *Energia z tłuszczu*	--	30.8 (27.1-33.7)	30.1 (27.1-33.0)	31.9 (29.6-35.0)	30.4 (26.3-33.3)	--	--	--	--
Energy from carbohydrates [%]^a^ *Energia z węglowodanów*	--	54.1 (50.6-58.6)	56.6 (52.6-59.5)	51.2 (48.6-54.5)	53.9 (50.3-57.8)	--	--	--	--
Energy from Saccharose [%]*^a^ Energia z sacharozy* ^a^	<10	13.5 (10.0-16.9)	11.4 (8.9-13.9)	13.9 (10.3-16.1)	14.7 (11.0-18.6)	26.0	36.2	21.2	20.7
Sodium [mg]^a^, ^b^ *Sód*	750+	1611.4 (1206.3-2072.3)	1280.9 (886.2-1639.5)	1851.8 (1669.0-2167.7)	1762.9 (1347.1-2170.3)	6.4	15.5	0.0	2.4
Potassium [mg]^a^, ^b^ *Potas*	2400+	1811.5 (1430.3-2163.4)	1561.3 (1329.9-2002.3)	2127.6 (1941.7-2393.9)	1810.5 (1446.9-2258.8)	85.5	96.6	75.8	81.7
Calcium [mg]^a^, ^b^ *Wapń*	500	561.1 (421.6-746.8)	568.7 (488.8-726.4)	855.9 (783.9-974.9)	451.5 (362.0-572.2)	38.7	29.3	3.0	59.8
Phosphorus [mg]^a^ *Fosfor*	380	745.2 (591.8-897.6)	650.4 (542.6-815.6)	1012.6 (874.1-1118.1)	693.1 (556.0-832.0)	4.0	5.2	0.0	4.9
Magnesium [mg]^a^ *Magnez*	65	159.0 (123.4-197.7)	135.1 (109.5-170.9)	195.8 (176.3-209.4)	157.6 (127.0-197.1)	1.7	1.7	0.0	2.4
Iron [mg]^a^ *Żelazo*	3	7.0 (5.6-9.0)	9.0 (7.7-10.6)	6.0 (5.0-6.9)	6.2 (4.9-7.8)	0.6	0.0	0.0	1.2
Zinc [mg]^a^ *Cynk*	2.5	5.9 (4.9-6.9)	6.4 (5.6-8.1)	6.2 (5.3-6.8)	5.1 (4.1-6.4)	1.2	0.0	0.0	2.4
Copper [mg] *Miedź*	0.25	0.6 (0.5-0.7)	0.6 (0.5-0.7)	0.6 (0.5-0.7)	0.6 (0.5-0.7)	0.0	0.0	0.0	0.0
Manganese [mg]^a^ *Mangan*	--	1.7 (1.2-2.3)	1.3 (0.9-1.8)	1.9 (1.5-2.1)	1.8 (1.3-2.5)	--	--	--	--
lodine [µg]^a^, ^b^ *Jod*	65	91.3 (68.9-114.9)	114.6 (94.3-135.5)	80.6 (67.3-100.1)	80.5 (59.8-99.5)	19.7	3.4	18.2	31.7
Vitamin A [µg] *Witamina A*	280	875.9 (583.7-1183.7)	923.9 (685.4-1191.4)	959.4 (688.5-1130.5)	805.1 (516.1-1173.8)	1.7	0.0	0.0	3.7
Vitamin E [mg]^a^, ^b^ *Witamina E*	6+	5.6 (4.0-7.6)	7.6 (6.6-9.3)	4.5 (3.8-6.0)	4.6 (3.5-5.9)	55.5	17.2	72.7	75.6
Thiamine [mg]^a^ *Tiamina*	0.4	0.8 (0.6-1.0)	0.9 (0.7-1.2)	0.9 (0.7-1.1)	0.7 (0.5-0.9)	3.5	3.4	0.0	4.9
Riboflavin [mg]^a^ *Ryboflawina*	0.4	1.2 (1.0-1.5)	1.2 (0.9-1.4)	1.7 (1.5-2.1)	1.0 (0.8-1.4)	0.0	0.0	0.0	0.0
Niacin [mg] *Niacyna*	5	9.4 (7.4-11.8)	9.8 (7.2-11.6)	9.5 (7.7-12.6)	9.0 (7.5-11.4)	6.4	6.9	0.0	8.5
Vitamin B6 [mg]^a^ *Witamina B6*	0.4	1.2 (0.9-1.4)	1.0 (0.8-1.3)	1.3 (1.0-1.7)	1.2 (0.9-1.4)	0.6	0.0	0.0	1.2
Vitamin B12 [µg]^a^ *Witamina B12*	0.7	2.2 (1.5-2.8)	1.8 (1.3 2.1)	3.3 (2.8-4.2)	2.2 (1.5-2.7)	1.2	0.0	0.0	2.4
Vitamin D [µg]^a^, ^b^ *Witamina D*	10	3.5 (1.6-6.2)	6.7 (5.6-8.9)	2.2 (1.2-3.8)	1.9 (1.2-3.1)	93.1	81.0	100.0	98.8
Vitamin C [mg]^a^ *Witamina C*	30	81.4 (56.1-112.6)	100.6 (77.3-124.6)	66.5 (51.4-86.8)	72.8 (44.4-109.5)	4.0	0.0	6.1	6.1
Folate [µg]^a^ *Foliany*	120	162.6 (132.8-199.9)	182.5 (156.3-219.5)	144.7 (128.6-180.8)	155.1 (126.9-186.1)	15.6	6.9	18.2	20.7
Folic acid [µg]^a^ *Kwasfoliowy*	--	3.9 (0.0-38.6)	42.3 (27.7-59.5)	0.0 (0.0-9.3)	0.0 (0.0-10.6)	--	--	--	--

Median (1Q-3Q)/mecf/ono *(1Q-3Q) -* median and interquartile *range/mediana i rozstęp kwartylny* EAR - Estimated Average Requirement/*Szocowoni*e *przeciętne zapotrzebowanie;* Al - Adequate Intake*/Wystarczajqce spożycie;* LCPUFA - Long chain polyunsaturated fatty *acids/Długołańcuchowe wielonienasycone kwasy tłuszczowe (DWKT)*

a Statistical significant differences in consumption of food products between the three clusters of children (Kruskal-Wallis rank Anova; *p<0.05)/Statystycznie istotne różnice w spożyciu produktów spożywczych pomiędzy trzema skupieniami dzieci (Anova rang Kruskala-Wallisa; p<0,05)*.

b Statistically significant differences in the odds of children from different clusters with consumption of food exceeding recommendations (chi2 test; *p<0.05)/Statystycznie istotne różnice w odsetkach dzieci z różnych skupień ze spożyciem żywności powyżej zaleceń (test chi^2^; p<0,05)*.

The fulfilment of the requirement for energy (EER) and fat (EAR) in the diets of children with different dietary patterns varied significantly. In the second cluster (“milk and cereals diet”) 93.9% of the toddlers exceeded the

Dietary patterns in toddlers with excess weight 275 requirement for energy (compared to 58.6% in the first cluster and 65.6% in the third cluster), 75.8% exceeded the requirement for fat (compared to 36.2% in the first cluster and 51.2% in the third cluster). Children from the second cluster did not fulfil the requirement for dietary fibre with significantly higher frequency than children from other clusters.

The diets of the majority of the children did not have any iron deficiencies or any deficiency of phosphorus, magnesium, zinc, copper and vitamin A, vitamins from the B group and vitamin C.

Nutrient profiles of the diets of children from all clusters exhibited significant deficiencies of LCPUFA and vitamin D. A deficiency of vitamin E was found in 72.7% and 75.6% of the children (cluster two and three), and potassium deficiency was observed in all the clusters (81.7-96.6%).

The requirement for calcium was fulfilled in 97.0% of the children in the second cluster, in 70.7% - in the first cluster and 40.2% of the children in the third cluster. Calcium deficiency was observed in 29.3% and 59.8% of the children (first and third cluster, respectively). Iodine intake was lower than the recommended level in 18.2% of the children from the second cluster and in 31.7% of the children from the third cluster.

Excessive sodium intake was found in the diets of all the children from the second and third cluster and in 85% of the children from the first cluster.

Nutritional practices in all the children analysed differ from the safe nutrition model – 79.3-89.0% of the children received snacks between meals every day or at least 2-4 times a week, had meals before bedtime (69.7% in the second cluster, 56.9% in the first cluster and 48.8% in the third cluster; p=0.06) and ate or drank during night time (46.3% in the third cluster, 39.4% in the second cluster and 37.9% in the first cluster; p=0.03). Children from the first cluster significantly more frequently received foodstu$s intended for infants and toddlers (junior formula, baby cereals and gruels) (p<0.05). The odds of breastfed toddlers were significantly higher in the third cluster (p=0.005) (Table IV ).

**Table IV:** Nutritional practices in children (overweight and obese) aged 1-3 years with different dietary patterns. Tabela IV. Organizacja żywienia dzieci w wieku 1-3 lata z nadmiarem masy ciała różniących się wzorami żywienia.

Nutritional practices *Organizacja żywienia*	Toddlers with excess weight [%] consuming various types of foods everyday or at least 2-4 times a week *Odsetek dzieci z nadmiarem masy ciała spożywających różne posiłki codziennie lub przynajmniej 2-4 razy w tygodniu* *lub przynajmniej 2-4 razy w tygodniu*				P value *poziom* *istotności p*
	All children *Dzieci ogółem* (N=173)	Cluster 1 *Grupa 1* BABY FOOD DIET (N=58)	Cluster 2 Grupa 2 MILK AND CEREALS DIET (N=33)	Cluster 3 *Grupa 3* SANDWICH AND SUGAR DIET (N=82)	
Breastfeeding *Karmienie* *piersią*	10.4	3.5	3.0	18.3	0.005^*^
**Main meals *Posiłki zalecane***					
Breakfast *I* *śniadanie*	99.4	100.0	100.0	98.8	0.1
Second breakfast *II śniadanie*	93.6	98.3	87.9	92.7	0.4
Soup *Posiłek obiadowy – zupa*	96.0	96.6	93.9	96.3	0.9
Main course dish *Posiłek obiadowy – II danie*	92.5	93.1	90.9	92.7	0.9
Afternoon snack *Podwieczorek*	93.6	94.8	93.9	92.7	0.6
Supper *Kolacja*	99.4	98.3	100.0	100.0	0.7
**Additional meals *Posiłki dodatkowe***					
Bedtime meal *Posiłek przed snem*	55.5	56.9	69.7	48.8	0.06
Eating/drinking during the night *Jedzenie/picie w nocy*	42.2	37.9	39.4	46.3	0.03^*^
Snacking *Pojadanie*	85.5	79.3	87.9	89.0	0.2
**Feeding practices *Forma żywienia***					
Family meals *Posiłki stołu rodzinnego*	86.7	81.0	87.9	90.2	0.5
Separate meals prepared for the child *Posiłki przygotowywane osobno dla dziecka*	25.4	34.5	18.2	22.0	0.6
Meals based on baby food *Posiłki na bazie żywności gotowej dla niemowląt i małych dzieci*					
follow-on/junior formula *mleko modyfikowane*	40.4	87.9	21.2	14.6	<0.0001^*^
vegetable purees, soups *obiadki, zupki*	15.6	24.1	12.1	11.0	0.06
baby cereals *kaszki/kleiki*	36.4	46.6	33.3	30.5	0.01^*^
fruit purees *przeciery, deserki owocowe*	22.0	25.9	15.2	22.0	0.3
juices *soki*	30.1	36.2	30.3	25.6	0.8
baby teas *herbatki*	19.1	29.3	15.2	13.4	0.3
Ready-to-serve meals *posiłki przygotowywane poza domem*	9.2	5.2	0.0	3.7	0.8

* Statistically significant differences between three clusters of children (p<0.05)/Sstatystycznie istotne różnice pomiędzy trzema grupami dzieci.

## Discussion

The study evaluated the diets of overweight and obese children from a representative nationwide group and identified three dietary patterns with varying energy and nutritional value. The “baby food diet” in younger children was the most balanced one when compared with the safe nutrition model. Rose et al proved that the dietary patterns of children in infancy had an impact on their diet and risk of obesity at preschool age [14]. Infants whose diet was higher in fruit and vegetables at 9 months had higher fruit and vegetable intake also at 6 years of age. Similarly, infants with a dietary pattern characterized by foods high in energy density (French fries, sweet desserts) continued to have higher consumption of these foods at 6 years old, and had a higher prevalence of overweight (43%). Formula-fed infants had higher sugar-sweetened beverage intake and fewer fruit and vegetable intake at 6 years than breastfed infants. Another study which aimed at identifying the dietary patterns of infants in the first year of life showed that the main determinants of their variability were not only the mother’s education and age, but also the place of residence [15]. Our results confirm the influence of the educational level of not only the mothers, but of both parents, on the dietary patterns of children in post-infancy.

The results of many studies proved that widespread prevalence of excess body weight even in the early period of life is correlated with the intake of food with high energy density [2, 3, 7, 16].

It is supposed that lower quality diets, high in energy-dense, high-fat products and low in dietary fibre consumed in childhood and adolescence are associated with the risk of obesity, as they undermine innate appetite control, which may lead to greater energy consumption [17]. Such a dietary pattern was found in overweight and obese children in the third year of life (cluster 2 and 3).

Inappropriate food choices and portion sizes and a diet which is well-balanced in terms of nutrient profile may be related with an increased risk of obesity [18, 19, 20].

In the study conducted the most frequently used food products were identified in the diets of children with excess weight. In toddlers in their second year of life (cluster 1) the main products were foods for special nutritional purposes, intended for young children, such as junior formula, baby cereals and gruels. In children in the third year of life (cluster 2 and 3) the diet base was cow’s milk, breakfast cereals, groats and bread. All the overweight and obese children consumed excessive quantities of meat and meat products, as well as foods being sources of sugar (dairy desserts, sweet beverages and juices).

Research conducted in recent years indicates that food choices following the safe nutrition model guidelines ensure the proper energy and nutritional value of a child’s diet and decrease the risk of developing eating disorders and obesity [7, 21, 22, 23].

The assessment of diets of children residing in different countries, including the European Union, showed that excessive energy and nutrient intake (i.e. excess of protein) is a risk factor for developing childhood obesity [2, 24]. It was also found that the adequate intake of macronutrients, calcium, fibre, vitamin D is negatively correlated with the risk of childhood obesity. On the contrary, the increased intake of B vitamins (B1, B2, niacin, which may enhance fat synthesis), excessive consumption of sweet beverages being sources of mono- and disaccharides contribute to developing obesity [7]. In our study we observed an excessive intake of energy, protein, sodium, B vitamins and saccharose and an insufficient supply of calcium, fibre, vitamin D, vitamin E, LCPUFA, iodine and potassium in children’s diet in reference to nutritional recommendations.

The analysis of nutrient profiles in the diets of the overweight children pointed to the need to popularise the model of food rations among the parents of young children.

## Conclusions

The identified dietary patterns of overweight toddlers differ from the safe nutrition model in terms of product selection and nutrient profile.

Younger children with excess weight, with separate dietary patterns, require fast nutritional intervention to introduce proper food choices in their diets.

Nutritional education is required for parents or caregivers of overweight toddlers, with a particular focus on the group with a significantly lower level of education.
